# Sickness absence among municipal workers in a Brazilian municipality: a secondary data analysis

**DOI:** 10.1186/s13104-017-3116-5

**Published:** 2017-12-28

**Authors:** Ana Lucia M. Leao, Anadergh Barbosa-Branco, Marília D. Turchi, Ivan A. Steenstra, Donald C. Cole

**Affiliations:** 1 Institute of Tropical Pathology and Public Health, Federal University of Goias, Goiânia, GO Brazil; 2 Dalla Lana School of Public Health, University of Toronto, Toronto, ON Canada; 3 Institute for Work & Health, Toronto, ON Canada; 4 Instituto de Patologia Tropical e Saude Publica-IPTSP/UFG, Rua 235-s/n-Setor Universitário, Goiânia, Go CEP: 74605-050 Brazil

**Keywords:** Sickness absence, Sick leave, Work disability, Musculoskeletal diseases, Mental disorders, Injuries, Job tenure, Cohort studies, Occupational health, Occupational epidemiology

## Abstract

**Background:**

Sickness absence, work disability associated with illness or injury, is a major public health problem worldwide. Some studies have investigated determinants of sickness absence among workers with shorter job tenure, but have only focused on certain diagnostic groups. Although it is well established that job tenure has an inverse relationship with work injury rate, less is known about its association with sickness absence for other disorders. Therefore, this study aimed to investigate the risk factors for incidence and duration of sickness absence according to diagnosis over a 7-year period. A dynamic cohort consisting of all permanent civil servants hired from 2005 to 2011 by the Goiania municipality-Brazil. Data of certified sickness absences longer than 3 days were analyzed. The incidence density was calculated per 1000 person-years in each ICD-10 category. The association between sickness absence and socio-demographic and occupational characteristics was examined using negative binomial regression models.

**Results:**

18,450 workers, mean age of 32 years, accumulated 14,909 episodes of sickness absence. Overall, the incidence density was 234.6 episodes per 1000 person years. Diagnostic groups with the highest incidence density of sickness absences were injuries (49.1), musculoskeletal disorders (31.3) and mental disorders (29.2). Factors predicting any sickness absence were female gender, older age, low education, being a health professional, multiple jobs and full-time employment. Mental health disorders were more common among education professionals, musculoskeletal disorders among blue collar workers and injuries among inspection workers. Prolonged time on sick leave was associated with male gender, older age groups, low education and income, blue-collar workers, more than one job contract and full time employment.

**Conclusions:**

These findings demonstrate a substantial sickness absentee burden and they provide relevant information for targeting prevention and health promotion policies to the most vulnerable occupational groups.

**Electronic supplementary material:**

The online version of this article (10.1186/s13104-017-3116-5) contains supplementary material, which is available to authorized users.

## Background

Sickness absence, work disability associated with illness or injury, is a major global public health problem and affects a large proportion of the working population and their quality of life. Sickness absence contributes substantially to healthcare costs, insurance expenditures, loss of economic productivity, and increases the risk of disability pension and mortality [1, 2]. Sickness absence data have been used as global indicators of the health of working populations to track trends and to guide prevention strategies [3].

Over the past decade, an increasing number of studies have assessed the sickness absence profiles of workers. However, most studies have focused on populations in developed countries; less is known about the burden of sickness absence in developing countries, which have greater socio-economic inequalities. Musculoskeletal and mental disorders are the most common causes of sick leave in developed countries [1, 4–6]. By contrast, a population-based study using disability claims in Brazil found that the main causes of sickness benefits, both work and non-work related, were injuries [7].

There is strong evidence that socio-demographic characteristics [6–8], occupation and working conditions [9–13], and the type and scope of social insurance systems [14], are among the most important factors associated with the incidence and duration of sickness absence. Although not fully explored, another potential factor is being employed in multiple jobs. A recent study in the USA showed that there was a high risk of injury among workers who had more than one job [15].

Most cohort studies start the follow-up with an employed population with varying lengths of exposure to occupational risk factors. Long-term exposure seems to increase the risk of some disorders, especially musculoskeletal [16]. A history of sickness absence is also an important predictor for a new episode, which is not always easy to verify and control for in a population that is already employed [17, 18].

In a previous study, we found that almost half of the sick leave episodes were experienced by workers in their first year on the job [19], which aroused our interest to further investigate the risk factors for sick leave at this early stage of one’s career. Some studies have investigated determinants of sickness absence among workers with shorter job tenure [16, 20, 21], but have only focused on certain diagnostic groups, such as work injuries. Potential reasons why shorter job tenure is associated with an increased risk of injuries include lack of knowledge and job experience, inadequate safety training and higher job turnover, which can increase the vulnerability of new employees [20]. Although it is well established that job tenure has an inverse relationship with work injury rate, less is known about its association with sickness absence for other disorders.

The purpose of this paper was to investigate the risk factors for incidence and duration of sickness absence in a large cohort of healthy workers, who were followed from their first day of work in the municipality. This study design enabled us to examine the effects of age and years of service on socio-economic and working conditions, and to investigate the differences in morbidity profiles by occupational categories.

## Methods

A dynamic cohort was created from all permanent civil servants of the Goiania municipality who were hired between January 1st, 2005 and December 31st, 2011. The workers lived in the Goiania metropolitan area in central-western Brazil, which has a population of about two million. The municipality operates a full range of public sector services, which includes health, primary education, various inspection services and a range of blue and white collar jobs (see Additional file 1: Appendix A).

### Brazilian public sector worker employment and benefit scheme

Civil servants in Brazil are hired through a public selection process. Knowledge tests are completed, followed by pre-employment medical examinations. Prospective employees must present health conditions compatible with their future occupation. In this process, health conditions are assessed in accordance to a public protocol. The medical evaluation includes a review of the applicant’s medical history, physical examination and complementary exams. Some exams are common to all occupations (for instance, Thorax radiography and glucose level) and others are job-specific (for example, “Physical agility tests” for guards, and “Laryngoscopy” for teachers). Following the probationary period, the worker has lifetime job security. Most public sector workers are hired in their 20s and 30s, and remain until retirement (approximately 60 years of age for women and 65 for men), unless one commits a serious offense to the public service, such as corruption, which can be a reason for dismissal [22].

Municipal workers have their own social benefit plan with mandatory insurance coverage for sickness absence, family assistance, family allowance, maternity leave, disability pension, age-based retirement, and retirement based on the length of service [23]. When sick, municipal workers must report to their employers immediately. If they return to work within 3 days, the episode remains self-certified. After 3 days, the municipal occupational health service conducts a medical assessment to diagnose the underlying cause for the absence and assigns a code from the 10th International Classification of Diseases (ICD-10).

### Sickness absence data

Sickness absence data (start date, sick-leave diagnosis and duration), socio-demographic and occupational data were obtained from the Goiania municipal records. Sick leaves due to pregnancy, childbirth, and the postpartum period were excluded. The worker’s identification number was used for the database linkage.

### Ethics statement

The study was authorized by the Goiania municipality and approved by the Ethical Board of the Federal University of Goias-Brazil. The Ethical Board waived the requirement for informed consent (IC), because we used secondary data which were anonymized and it is not possible to contact the study subjects in order to obtain their IC.

### Covariates

The following explanatory variables were included in the study: gender, age (grouped), marital status classified as married or not married (widowed, never married, divorced or separated), level of education (categorized as low: up to 9 years of schooling, middle: up to 12 years, and high: 15 years or more), salary (tertiles of gross monthly income), number of job contracts (single or multiple), and work schedule [part-time or full-time (at least 40 h/week)]. Occupational classes were grouped into five categories (Additional file 1: Appendix A): (i) white collar (e.g., administrative assistant, economist), (ii) blue collar (e.g., cleaner, bricklayer), (iii) health professional (e.g., nurse, physician), (iv) inspection worker (e.g., municipal guard, parking enforcement), and (v) education professional (e.g., early or elementary school teacher). All of the variables that were included as risk factors were measured at the time of hiring.

### Data analysis

The outcome measure was medically certified sickness absence (longer than three consecutive days), which included the number of episodes and days of sick leave over the seven-year follow-up period. Sickness absences for the same diagnosis and within 3 days of the return-to-work were considered to be a continuation of the earlier absence, and were not considered to be new episodes. The population was followed from the first day of work until retirement, death, resignation or the end of the study period (December 31st, 2011), whichever came first.

The person-years at risk were calculated by subtracting the number of days of sick leave from the total length of follow-up. The incidence density of sickness absence was calculated by dividing the number of episodes in each ICD-10 category by the population at risk, and was expressed per 1000 person-years. We examined diagnostic categories that included more than 500 episodes of sickness absence during the follow-up period.

The association between sickness absence episodes and socio-demographic and occupational characteristics was examined using Negative Binominal regression models, with the logarithm of days at risk as the offset. Ninety-five percent confidence intervals (95% CIs) were computed for each rate ratio (RR). We studied sickness absences resulting from all medical causes and the three most common diagnoses. We also used Negative Binomial regression to analyze the number days of sick leave, and estimated RR (95% CIs) for each prognostic factor for duration of sickness absence.

All statistical analyses were performed using SAS statistical software, version 9.3.

## Results

A total of 18,450 civil servants, with a mean age of 32 years [standard deviation (SD) 9.0] contributed 65,157.75 person-years to the study population; the mean follow-up time per worker was 3.5 years (SD 1.7). Table 1 presents the baseline characteristics, the workers were mostly women (67.9%), aged 25–34 years (41.6%), not married (52.6%), with a middle education (47.6%), employed as education professionals (22.7%), with a single job contract (55.8%), and who worked part-time (67.8%) (Table 1).

**Table 1:** Demographic and occupational characteristics of the study population

Characteristics	N	%
Gender		
Male	5924	32.1
Female	12,526	67.9
Age group (years)		
≤ 24	3289	17.8
25–34	7667	41.6
35–44	5115	27.7
≥ 45	2379	12.9
Marital status		
Not married	9705	52.6
Married	8745	47.4
Education level (years)		
Low (≤ 9)	2400	13.0
Middle (10 to 12)	8786	47.6
High (> 12)	7264	39.4
Occupational class		
White collar	3840	20.8
Education	4188	22.7
Blue collar	3755	20.4
Health	3545	19.2
Inspection	3122	16.9
Number of job contracts		
One	10,299	55.8
Multiple	8151	44.2
Work schedule		
Part-time	12,515	67.8
Full-time	5935	32.2

Goiania municipality, Brazil; 2005–2011

### Incidence density of sickness absence

Over the 7 total years of follow-up, there were 14,909 episodes of sickness absence, totaling 323,646 lost work days. Overall, the incidence density was 234.6 episodes per 1000 person-years of observation; the median time to the onset of the first sickness absence was 1.5 years. In Table 2, the incidence density of sickness absence is ranked from the most common ICD-10 category to the least common. Injury, poisoning and other consequences of external causes (Injury, 49.1/1000 person-years) ranked the highest and were 1.5 times higher than the next two most common categories, including musculoskeletal disorders (31.3/1000 person-years), and mental health and behavioral disorders (29.2/1000 person-years). Injuries accounted for approximately 25% of the total number of days of sickness absence, followed by mental health (17.5%) and musculoskeletal disorders (13.9%) (Table 2).

**Table 2:** Incidence density and total duration of sickness absences according to main ICD-10 diagnostic categories (chapters)

Sickness absence diagnosis (ICD-10 codes)	N (%)	ID (CI 95%)	Total SA duration (%)
Injury and poisoning	3156 (21.2)	49.1 (46.8–51.5)	78,083 (24.1)
Musculoskeletal disorders	2027 (13.6)	31.3 (29.4–33.4)	45,011 (13.9)
Mental health and behavioural disorders	1832 (12.3)	29.2 (26.9–31.6)	56,480 (17.5)
Infectious and parasitic diseases	1069 (7.2)	16.4 (15.3–17.5)	9506 (2.9)
Diseases of the circulatory system	861 (5.8)	13.1 (12.1–14.1)	20,445 (6.3)
Diseases of the genitourinary system	857 (5.7)	13.1 (12.1–14.3)	7253 (2.2)
Diseases of the respiratory system	833 (5.6)	13.0 (12.0–14.1)	13,331 (4.1)
Disease of the digestive system	773 (5.2)	11.9 (11.0–12.9)	12,519 (3.9)
Disease of the eye and adnexa	667 (4.5)	10.3 (9.4–11.2)	6823 (2.1)
Neoplasms	504 (3.4)	7.7 (6.9–8.8)	23,134 (7.1)

Goiania municipality, Brazil; 2005–2011

*ID* incidence density: number of episodes of sickness absence in each ICD-10 groups/1000 person-years at risk

*CI* confidence intervals

*SA* sickness absence

### Risk factors for incidence and duration of sickness absence

Socio-economic and occupational differences in sickness absences due to all causes and by the three most common diagnoses are summarized in Table 3. Overall, women had a 32% higher occurrence of absences than men. The occurrence of absence increased with age. A lower level of education was consistently associated with a higher absentee rate (RR 1.48, CI 95% 1.33–1.65). Health professionals had high rates of absence (RR 1.49, CI 95% 1.36–1.63) compared with white collar workers. Having more than one job contract (RR 1.06, CI 95% 1.01–1.12) and full-time employment (RR 1.14, CI 95% 1.07–1.21) independently increased the occurrence of sickness absence.

**Table 3:** Rate ratios (RR) of sickness absence by socio-demographic and occupational characteristics for the three most common ICD-10 diagnoses

Explanatory variables	All causes	Injury	Musculoskeletal	Mental
	RR (CI 95%)	RR (CI 95%)	RR (CI 95%)	RR (CI 95%)
No. SA episodes	14,909	3156	2027	1832
Gender				
Male	1	1	1	1
Female	1.32 (1.23–1.41)	0.80 (0.71–0.90)	1.35 (1.14–1.59)	1.36 (1.10–1.67)
Age group (years)				
≤ 24	1	1	1	1
25–34	1.10 (1.02–1.19)	0.90 (0.79–1.04)	1.67 (1.35–2.07)	0.93 (0.74–1.18)
35–44	1.10 (1.02–1.20)	0.85 (0.73–0.99)	1.65 (1.31–2.07)	0.87 (0.67–1.13)
≥ 45	1.23 (1.11–1.36)	0.93 (0.77–1.12)	2.72 (2.11–3.51)	0.87 (0.64–1.19)
Marital status				
Not married	1	1	1	1
Married	0.98 (0.93–1.03)	0.85 (0.77–0.94)	1.02 (0.90–1.17)	0.78 (0.66–0.92)
Education level (years)				
Low (≤ 9)	1.48 (1.33–1.65)	2.18 (1.79–2.66)	2.10 (1.62–2.72)	1.16 (0.82–1.64)
Middle (10–12)	1.33 (1.23–1.44)	1.96 (1.74–2.19)	1.52 (1.25–1.86)	1.04 (0.81–1.32)
High (> 12)	1	1	1	1
Income tertiles				
Lowest	0.92 (0.85–1.01)	0.81 (0.69–0.95)	0.99 (0.80–1.24)	1.04 (0.78–1.37)
2nd	0.94 (0.87–1.02)	0.96 (0.84–1.11)	1.01 (0.83–1.23)	0.84 (0.65–1.08)
Highest	1	1	1	1
Occupational class				
White collar	1	1	1	1
Education	1.45 (1.31–1.60)	1.68 (1.38–2.03)	1.71 (1.32–2.21)	1.40 (1.03–1.90)
Blue collar	1.32 (1.20–1.40)	1.56 (1.31–1.85)	1.73 (1.38–2.17)	0.97 (0.73–1.29)
Health	1.49 (1.36–1.63)	1.57 (1.31–1.87)	1.44 (1.13–1.83)	1.21 (0.91–1.60)
Inspection	1.31 (1.19–1.43)	1.81 (1.53–2.13)	1.36 (1.08–1.73)	0.85 (0.63–1.13)
Number of job contracts				
One	1	1	1	1
Multiple	1.06 (1.01–1.12)	0.93 (0.84–1.02)	1.13 (0.99–1.29)	1.34 (1.13–1.59)
Work schedule				
Part-time	1	1	1	1
Full-time	1.14 (1.07–1.21)	1.16 (1.03–1.30)	1.10 (0.94–1.29)	1.15 (0.96–1.39)

Goiania municipality, Brazil; 2005–2011

*RR* rate ratio, *CI* confidence interval, *SA* sickness absence

When examining the factors associated with the most common ICD-10 diagnoses for sickness absences (injury, musculoskeletal and mental disorders), women had significantly higher rates of sickness absence related to mental and musculoskeletal disorders, while men did for injury (see Table 3). The risk of absence due to musculoskeletal disorders increased with age (RR varying between 1.67 and 2.72 depending on the age group); by comparison, the age range of 35–44 years was a protective factor against the risk of injuries. Being married reduced the risk of injury (RR 0.85, CI 95% 0.77–0.94) and mental disorders (RR 0.78, CI 95% 0.66–0.92). Lower education doubled the risks of absence for injuries (RR 2.18, CI 95% 1.79–2.66) and musculoskeletal disorders (RR 2.10, CI 95% 1.62–2.72). Blue-collar workers had a 1.73 times higher risk of musculoskeletal diseases compared with white-collar workers. Inspection workers had an 81% higher occurrence of injuries compared with white-collar workers. Those who were more than one job contract (RR 1.34, CI 95% 1.13–1.59) and education professionals (RR 1.40, CI 95% 1.03–1.90) had higher risks of mental health-related SAs. Finally, working full-time increased the risk of injuries.

Table 4 presents the relationship of each factor under study with the sickness absence duration. Female gender, worker’s age (up to 44 years), and higher education were associated with a shorter sick leave duration. An increase in sickness absence duration was observed among workers with lowest income (RR 1.12), and blue-collar in comparison to white-collar workers. Furthermore, more than one job contract (RR 1.15) and full-time employment (RR 1.09) were also associated with a longer duration.

**Table 4:** Prognostic factors for duration of sickness absence

Prognostic factors	Rate ratio (95% CI)	*P* value
Gender		
Female	0.92 (0.86–0.98)	0.0095
Male	1	
Age groups		
≤ 24	0.68 (0.62–0.75)	< 0.0001
25–34	0.79 (0.74–0.86)	< 0.0001
35–44	0.88 (0.81–0.95)	0.0015
≥ 45	1	
Marital status		
Married	0.03 (0.98–1.08)	0.2983
Not married	1	
Education level		
Low	0.05 (0.97–1.13)	0.2318
High	0.92 (0.86–0.99)	0.0353
Middle	1	
Income tertiles		
Lowest	1.12 (1.03–1.22)	0.0061
2nd	0.99 (0.92–1.06)	0.7255
Highest	1	
Occupational class		
Blue collar	1.11 (1.02–1.21)	0.0178
Education	1.03 (0.93–1.13)	0.5770
Health	0.01 (0.92–0.10)	0.8870
Inspection	0.06 (0.97–1.16)	0.1950
White collar	1	
Job contracts		
Multiple	1.15 (1.09–1.21)	< 0.0001
One	1	
Work schedule		
Full-time	1.09 (1.03–1.16)	0.0025
Part-time	1	

Goiania municipality, Brazil; 2005–2011

## Discussion

The present study found a high incidence of sickness absence among public sector workers early in their careers (234.6 episodes per 1000 person-years of observation). It is worth noting that this is a cohort of young adults employed after receiving medical certification of good health. There are several reasons for the increased risk of sickness absences among workers with shorter job tenure, such as, lack of job experience and knowledge, failures in training systems, low supervisor support, and difficulties in adapting quickly to the new work environment and tasks [20, 21]. These reasons can expose workers to high levels of job strain, causing them to take more sick leave as a coping mechanism to deal with work-related stress [13, 24]. In addition, these workers may experience greater job-anxiety due to performance standards and expectations that must be fulfilled during probation period, which can raise the risk of sick leave. According to Muschalla and Linden [25] workplace-related anxieties are often connected with sickness absence. Lastly, employees, especially younger ones, may be less committed to the organization and face the burden of ongoing educational demands and its effects on their private life [17].

The incidence of sickness absence among municipal public sector workers in the present study was 5.4 times higher than among employees in the private sector in Brazil (42.1 per 1000 person-years) [7]. However, comparisons between public and private sector sick leave studies should be made with caution. First, there are methodological differences between these studies, especially the requirement for medical certification; the time frame is 3 sick days in the public sector versus 15 sick days in the private sector. Furthermore, civil servants seem to be on sick leave more frequently than other workers, which could partly be caused by sickness absence policies; public sector insurance systems seem be more generous than those in the private sector [26].

Although municipal public sector workers presented a higher incidence of sick leave, the morbidity profile was similar to that described for Brazilian private sector workers [7], with a predominance of injuries, musculoskeletal and mental disorders. The latter two causes are also the most common causes of sick leave in developed countries [1, 4–6], while injuries seem to have a minor impact on the absentee burden in developed countries. Studies from the Netherlands [5] and the UK [6] showed an incidence of sick leave due to injuries ranging from 7.7 to 19.6 per 1000-person years. In contrast, in the present study, injuries were the most common cause of sick leave, yielding an incidence of 48.3 per 1000-person years.

While sick leave due to injury has decreased in most developed countries [10, 27], the incidence remains high in Brazil. This difference is likely due to the limited implementation of safety measures and ineffective enforcement of labour regulations. Moreover, the inefficiency of urban mass transportation has increased the number of bicycles and motorcycles on public roads, which, added to the lack of traffic education, can raise the risk of injuries [28].

In developed countries, injuries are the third most common cause of death in people between the ages of 5 and 44 years [29]. By comparison, injuries are the leading cause of death for the same age range in developing countries, due mainly to interpersonal violence and traffic collisions, particularly among men [27]. In Brazil, injuries due to traffic accidents have a strong impact on morbidity and mortality [28]; workers are known to have similar illness profiles as the general population, which was confirmed by our results.

The higher incidence of sickness absences among women, which was observed for all diagnostic groups except injury, has been previously described in cohort studies from the UK [6], France [1], Finland [11], and Brazil [7]. Higher results for women seem to be influenced by a combination of biological, psychosocial and cultural factors, that range from greater physical vulnerability; multiple social roles, with combined job and family responsibility; to sex segregation within the labour force [30]. The higher risk of mental and musculoskeletal disorders among women has been observed in other studies [1, 7, 11], partly as a result of sex segregation in different jobs. In general, women work mainly within the health, cleaning, educational and clerical services, which are characterized by high physical and emotional demands, lack of autonomy, low wages, social support and career perspectives that lead to a higher risk of sick leave [30].

The higher risk of injury among men, especially younger men, is also seen in results from previous study [10] and may be due to the lack of work experience and professional qualifications [21], and urban violence. Behavioral issues more prevalent in males, such as the consumption of alcohol and drugs mixed with driving, may contribute to the high incidence of injuries arising from traffic accidents, especially among relatively inexperienced drivers [27].

The association between incidence and longer duration of sickness absence and aging, especially due to musculoskeletal disorders, is consistent with the literature [7, 31, 32]. It is believed that the decline in physical work capacity, on average 20% between 40 and 60 years, is associated with cumulative effects of exposure to several occupational risk factors, which can increase the risk of injury and illness over prolonged years of working [33].

The higher risk of sickness absences among workers with lower levels of education is consistent with previous studies [10, 34]. Better education may provide workers with greater knowledge in preventive health measures, encourage the adoption of safer workplace practices, provide better career opportunities, facilitate increased access to health care and lead to healthier lifestyles and behaviors [34].

In this study, we assumed that occupational class, the number of job contracts and work schedules were indicators of workload and working conditions. The higher risk of incidence and longer duration of absences among civil servants with more than one job and with full-time employment reinforces the relationship between high workload and illness, attributable to fatigue from long hours, working multiple shifts, sleeping less and having additional stress. It also leads to deteriorating physical and mental health and may contribute to absenteeism [15, 35].

The increased risk of sick leave among health professionals is consistent with previous studies [9, 26, 36], whereby employment in human services involves an exposure to high emotional demands and to adverse working conditions such as irregular work hours, tight schedules, lack of autonomy, low job control, and inadequate support from colleagues and supervisors [12].

Certain professions are more prone to injury due to environmental and labour risk factors. In this regard, the higher risk of injury among inspection workers may be related to exposure to more hazardous conditions due to outdoor work activities. These workers are responsible for monitoring and supervising public safety, traffic enforcement, inspection or surveillance of public assets, which could increase their susceptibility to traffic-related injuries, urban violence, threats, and abuse.

The higher incidence of mental health disorders among teachers may be a result of working with people and being engaged in activities with high emotional demands, which increase the risk of psychiatric sickness absences, as was shown by British [26], Dutch [9] and Danish [37] studies. The latter study suggested that teachers display a high degree of commitment despite the lack of rewards they receive for their work (salary, career opportunities, and status), increasing demands, and limitations in their jobs. In Brazil, teachers are also exposed to workplace violence, threats, and abuse within the school environment, which increase work-related stress and may contribute to sick leave from psychiatric disorders [38].

The higher risk of incidence and longer duration of sick leave due to musculoskeletal disorders among blue collar workers is in line with previous studies [7, 12, 26, 32]. Manual workers are more susceptible to adverse physical working conditions [12], such as exposure to awkward postures, carrying heavy loads, experiencing vibrations and torsion movements of the trunk, which are related to the development of back pain and other musculoskeletal complaints, which can increase sickness absence.

### Study strengths and limitations

One of the main strengths of this study is that it analyzed the entire population of public sector workers from a municipality across all occupations in a range of workplaces and followed them for 7 years. According to a rigorous medical examination, all of the employees were healthy when they began to work. The risks of reporting bias, missing information and loss to follow-up were minimized because official administrative municipal databases were used. Another strength is that the outcome data (certified sickness absence) were derived from medical registers, rather than self-reports. Official medical registers provide accurate diagnosis-specific sickness absences and reduce the risk of incorrectly estimating the number and the duration of sickness absences.

The main limitation of this study is that it was not possible to distinguish work-related from non-work related morbidity, making it difficult to analyze the impact of working conditions on specific causes of sickness absences. Our study did not discriminate between short and long-term sick leave, as these have potentially different severity and socio-economic repercussions. We should point out that the burden of sickness absences may be underestimated, since episodes of sick leaves of up to 3 days/month are reported only at the workplace. Therefore, these non-certified short-term sickness absence could express much higher indicators. Another methodological limitation of the study is that the socio-demographic and occupational characteristics were not updated annually but were retained from the time of hiring. We recommend that future studies address these aspects and analyze the working conditions through their physical and psychosocial dimensions.

## Conclusions

Sickness absence due to injuries was confirmed as an important public health problem in developing countries, especially among young adults. We found clear differences in risk factors for sickness absences between the diagnostic groups. Our results indicate a morbidity profile according to occupational class, whereby education workers presented more risk of sick leave due to mental health disorders, blue collar due to musculoskeletal disorders, and inspection and health workers due to injuries. This study provides relevant information for disease prevention and health promotion policies. Special attention should be paid to employees in the beginning of their career, prioritizing the allocation of public health resources to the most vulnerable occupational groups. Health promotion strategies must be sensitive to the specific characteristics of subgroups and morbidity profiles; they should improve physical and psychosocial working conditions and adopt better and safer practices, aimed at reducing sickness absences.

## Additional file

**Additional file 1: Appendix A.** Broad occupational classifications of public sector job titles. Table containing the description of the job titles according to the five occupational classes studied.

## Abbreviations

SA: sickness absence
ICD-10: 10th International Classification of Diseases
95% CIs: ninety-five percent confidence intervals
RR: rate ratio
SAS: State-of-the-art Statistical Analysis software
SD: standard deviation
