# Supplementary material for: Sickness absence among municipal workers in a Brazilian municipality: a secondary data analysis
Source: BMC Res Notes. 2017 Dec 28;10:773. doi: 10.1186/s13104-017-3116-5 (PMC5745616; doi:10.1186/s13104-017-3116-5)
Supplement: Supplementary file 1 — Additional file 1: Appendix A. Broad occupational classifications of public sector job titles. Table containing the description of the job titles according to the five occupational classes studied. [file 13104_2017_3116_MOESM1_ESM.docx]

**Appendix A**

Broad occupational classifications of public sector job titles

| **Health** | **Blue Collar** | **White Collar** |
| --- | --- | --- |
| Art therapist | Assistant of food services | Accountant |
| Audiologist | Bricklayer | Administrator |
| Biochemist | Carpenter | Archivist |
| Dental assistant | Cemetery caretaker | Assistant administrative |
| Dentist | Charger | Attorney |
| Epidemiologist | Cleaner | Clerk Typist |
| Laboratory Technologist | Cook | Economist |
| Microbiologist | Driver / School bus driver | Librarian |
| Music therapist | Electricians | Manager |
| Nursing Assistant | Equipment Operator | Receptionist |
| Nutritionist | Gardener | Secretary |
| Occupational Therapist | Heavy equipment operator | Systems analyst |
| Pharmacists | Locksmith | Tax collector |
| Physician | Machine operator | Others |
| Physiotherapist | Maintenance repairer |  |
| Psychiatrist | Painter | **Inspection** |
| Psychologist | Plumber | Environmental inspector |
| Registered Nurse | Porter | Food Inspector |
| Sanitarian | Zoo animal keeper | Municipal guard |
| Social Worker | Others semi-skilled laborers | Park Ranger |
| Veterinarian |  | Parking enforcement |
| Others | **Education** | Sanitation Inspector |
|  | Early childhood teacher | Traffic control |
|  | Elementary school teacher | Others – Fiscal Occupations |
